# Rationally tailored antibiotic selection circumvents toxin-mediated evasion of killing in *Mycobacterium abscessus*

**DOI:** 10.3389/ftubr.2026.1921029

**Published:** 2026-10-27

**Authors:** Nikita H. Amin, Eduardo A. Troian, Nancy A. Woychik

**Affiliations:** Department of Biochemistry and Molecular Biology, Rutgers Health—Robert Wood Johnson Medical School, Piscataway, NJ, United States

**Keywords:** DNA gyrase, erm41, intrinsic resistance, persistence, ribosome, RNA polymerase, sigH, tRNA

## Abstract

Among clinically important non-tuberculous mycobacteria, *Mycobacterium abscessus* (Mab) has a large and highly dynamic accessory genome within an open pangenome, contributing traits that enhance drug resistance, bacterial persistence, stress survival, metabolic flexibility, and/or virulence. Defining how accessory loci promote survival during antimicrobial exposure is essential for understanding why Mab frequently evades killing by standard drug regimens and for improving therapeutic outcomes. We recently identified a potent accessory toxin–antitoxin (TA) system, VapB5-VapC5, that substantially enhances the already formidable drug resistance capacity of Mab clinical isolates. Acquisition of this single system reprograms Mab physiology to inhibit growth, activate the WhiB7 regulon, and promote ribosome accumulation. Here, we further define the contribution of the active component of this TA system, the VapC5 toxin, by assessing its impact on a more comprehensively defined WhiB7 regulon. We also studied the effects of VapC5 on growth, persistence, and the Mab transcriptome independently of WhiB7. Finally, we determined that VapC5-mediated resistance is mechanism-dependent. Because our previous study showed that VapC5 promotes survival during exposure to frontline antimicrobials that target cell-wall biosynthesis or protein translation, we reasoned that antibiotics with distinctly different mechanisms of action would be more likely to retain activity. As predicted, DNA gyrase or RNA polymerase inhibitors circumvented VapC5-mediated resistance and retained *in vitro* efficacy. These findings establish VapC5 as a broad physiological modulator that selectively reshapes Mab antimicrobial susceptibility based on drug mechanism. Screening Mab clinical isolates for acquisition of this accessory VapBC5 toxin-antitoxin system could risk-stratify treatment decisions by directing clinicians toward existing antibiotics targeting processes unaffected by VapC5, thereby improving treatment outcomes.

## Introduction

Among non-tuberculous mycobacteria, *Mycobacterium abscessus* (Mab) causes the majority of infections within the Mab complex and is notably the most resistant to clearance after aggressive and lengthy antimicrobial therapy. The intrinsic antibiotic resistance of Mab has been attributed to activation of transcription factor WhiB7 and upregulation of its regulon, which engage multiple disparate pathways toward resistance (1, 2). We recently reported that acquisition of a single VapBC5 toxin–antitoxin (TA) system further amplifies resistance in clinical strains (3). VapBC5 is a type II TA system whose general function is to facilitate stress survival after the toxin is released from its cognate antitoxin, allowing the toxin to act on its cellular target(s) [reviewed in (4)]. Toxin activity is generally thought to compromise antibiotic efficacy because toxin expression can promote the formation of non-dividing persister cells that are refractory to many antibiotics [reviewed in (5–7)].

We have previously shown that the VapC5 toxin specifically cleaves (and depletes) one tRNA^Ser^ species, inducing growth arrest and reprogramming physiology by favoring the synthesis of proteins encoded by transcripts with low serine codon content (3). Consequently, this adaptation enables Mab to persist in culture after exposure to antibiotics that primarily target actively dividing cells. In addition, VapC5 enhances expression of ribosomal proteins, whose transcripts have low serine codon content, thereby promoting survival after exposure to ribosome-targeting antibiotics (3). VapC5 also upregulates the *whiB7* gene and its regulon in the absence of antibiotic exposure (3).

Here, we conducted follow-up studies to further define how VapC5 reshapes Mab physiology and to test alternate antibiotics predicted to retain activity against VapC5-expressing strains. First, we assessed the effects of VapC5 expression on a more comprehensively defined WhiB7 regulon since our previous study examined only five well-defined regulon members. Second, to distinguish WhiB7-dependent from WhiB7-independent VapC5 effects, we expressed VapC5 in a *ΔwhiB7* background and examined changes in global transcriptional profiles, with particular emphasis on upregulated transcripts and the SigH regulon. Third, we tested whether WhiB7 is required for VapC5-mediated toxicity or persistence. Finally, we tested the *in vitro* efficacy of antibiotics, with mechanisms of action predicted to circumvent VapC5-mediated reprogramming. We identified DNA gyrase and RNA polymerase inhibitors as promising therapeutic options for clinical strains that have acquired the VapBC5 TA system.

## Material and methods

### Strains, plasmids, and reagents

All experiments utilized *M. abscessus* L948 (ATCC 19977) grown in 7H9 Middlebrook medium containing 0.05% Tween 80, 0.5% BSA, 0.2% dextrose, 0.085% NaCl, 0.1% casamino acids, and 25 µg/mL kanamycin (for plasmid selection). The cultures were grown with glass beads at 37°C under constant agitation. The anhydrotetracycline (ATc) inducible pMC1s-*vapC5* plasmid was described previously in Troian et al. (3) and transformed by electroporation into competent Mab ATCC 19977 cells. VapC5 expression was induced by adding ATc to the media to a final concentration of 200 ng/mL when cells reached an OD_600_ of ∼0.1. ATc was also added to the empty plasmid control when cultures reached an OD_600_ of ∼0.1.

### Construction of the *ΔwhiB7* mutant

The whiB7 deletion mutant (*ΔwhiB7*) was generated using oligonucleotide-mediated recombineering followed by Bxb1 integrase targeting (ORBIT), as previously described by Murphy et al. (8). A 188-mer polyacrylamide gel electrophoresis (PAGE)-purified single stranded DNA oligonucleotide (NWO3671, 5ʹ-TGCTCCCCATCGCCGAATGACGATGGGGAGCAATGGTTATCAGGCCCTACAACCTTCTTGGCCGGCGGCGGGTTTGTACCGTACACCACTGAGACCGCGGTGGTTGACCAGACAAACCGTGTCCGTTCACCTGCTTCCTCTTCGGTTCCTCGTAAAACTGCTGGTCTCGTATCGGTGTCTTATCGATC-3ʹ) was designed as the ORBIT targeting oligonucleotide and used to direct deletion of *whiB7*. Putative recombinants were first screened by PCR using primers flanking the *whiB7* gene (NWO3673 is the 5ʹ flanking oligonucleotide, 5ʹ-TTACGGAGACACCTTGTGGC-3ʹ and NWO3672 is the 3ʹ flanking oligonucleotide, 5ʹ-GATCGCCGAACAATGGATGC-3ʹ) to confirm its substitution by the linearized pKM496 plasmid flanked by *att*L/*att*R sites. Successful deletion of *whiB7* was further confirmed by the presence of the expected amplicon sizes using the *whib7* 5ʹ oligonucleotide (NWO3673) combined with *Zeo^R^*- (NWO3636, 5ʹ-GAATGCATCTCTCCCCATGCGAGAGTAG-3ʹ or *oriE*-specific (NWO3637, 5ʹ-GGAAACGCCTGGTATCTTTATAGTCCTG-3ʹ) primers to determine insert orientation. RNA-seq also confirmed the absence of *whiB7* and the lack of upregulation of regulon members.

### RNA isolation

Total RNA was isolated from ∼20 mL of Mab + VapC5 cells in each induction/treatment condition [(1) -ATc -rifabutin (RFB); (2) -ATc + RFB; (3) +ATc -RFB; (4) +ATc + RFB]. Cultures were grown in 125 mL of 7H9 Middlebrook medium with the supplements and conditions listed above until the OD_600_ reached ∼0.1. The cultures were then split into two 60 mL cultures. Expression of VapC5 was induced with ATc in just one culture. The cultures were then allowed to grow for another 5–6 h. After the induction period, the two sets of cultures were further split into 25 mL cultures. Of these, one culture was left untreated while the other culture was treated with 0.25 µg/mL of RFB for both the induced and uninduced cultures. The cultures were allowed to grow for another 30 min before the cells were collected at 2,300×*g* at 4°C for 15 min. The supernatant was discarded, and the cell pellets were resuspended in 1 mL of TRIzol reagent (Zymo Research) and transferred to 2 mL lysing kit tubes (Bertin Corp.) containing 0.1 mm beads. The Precellys Evolution homogenizer (Bertin Corp.) was used to lyse the cells using three 30 s 9,000 rpm cycles with 1 min cooling periods between each cycle. The lysate was centrifuged for 5 min at 16,000×*g* at 4°C, and RNA was extracted from the supernatant utilizing the Direct-zol RNA MiniPrep Plus Kit (Zymo Research). The RNA was treated with 1 U of Turbo DNase (Invitrogen) for 1 h at 37°C and then purified utilizing the RNA Clean and Concentrator Kit (Zymo Research). The RNA concentrations were measured using a Qubit 4 (ThermoFisher).

### RNA-Seq and DAVID analysis

To remove 16S and 23S ribosomal RNA (rRNA) from total RNA extractions, samples were processed using the rRNA depletion protocol described previously (3). The extracted RNA was incubated with biotinylated oligonucleotides designed to hybridize to bacterial 23S, 16S, and 5S rRNAs. The resulting biotinylated RNAs were captured using streptavidin-coated magnetic beads. Subsequently, 100 ng of rRNA-depleted RNA was used for library preparation using the NEBNext Ultra II Directional RNA Library Prep Kit for Illumina (New England Biolabs) and libraries were sequenced on an Illumina HiSeq 4000 platform. The resulting sequencing reads were aligned to the *M. abscessus* reference genome (NCBI accession CU458896.1) using Bowtie2 v2.5.4 default parameters. StringTie v1.3.4 and Limma-Voom v3.83.3 programs were used for transcript assembly and differential gene expression analysis, respectively. Gene-level read counts were determined using featureCounts and normalized using the trimmed mean of *M*-values. Differential expression was assessed using limma-voom with weighted linear models followed by empirical Bayes moderation. *p*-values were then adjusted for multiple testing using the Benjamini–Hochberg method.

Functional enrichment analysis was determined using the Database for Annotation, Visualization and Integrated Discovery (DAVID). Enrichment was assessed using their modified Fisher's exact test, and *p*-values were determined using the Benjamini–Hochberg method. Categories with an adjusted *p*-value ≤0.05 were considered significantly enriched.

### cDNA synthesis and qPCR

cDNA was synthesized from isolated RNA utilizing SuperScript™ IV Reverse Transcriptase (Invitrogen) and 50 µM random hexamers. The reactions were performed using the manufacturer's protocol. QuantStudio™ 3 (ThermoFisher) was utilized to perform real-time quantitative PCR (qRT-PCR) with the synthesized cDNA. qRT-PCR amplification was performed with PowerUp™ SYBR™ Green Master Mix for qPCR (ThermoFisher) using 1 µL of the final cDNA derived from 88 ng of total RNA. The reactions were performed using the manufacturer's protocol. The PCR conditions were 95°C for 10 min, followed by 40 cycles of 95°C for 15 s and 60°C for 60 s. Three technical and three biological replicates were used for each condition. The following primer pairs were utilized: *sigA* (NWO3525 5ʹ-CACATGGTCGAGGTCATCAA-3ʹ and NWO3526 5ʹ-TGGATTTCCAGCACCTTCTC-3ʹ), *whiB7* (NWO3515 5ʹ-CCTGTGGTTCGCGGAAA-3ʹ and NWO3516 5ʹ-CCCTGCTC AAGAATCTCACC-3ʹ), and *erm(41)* (NWO3517 5ʹ-GCACTGATACGGAGTCTCTTG-3ʹ and NWO3518 5ʹ-CTCGCTTCGCATGTTTGTG-3ʹ). The specificity of the primer pairs was confirmed in Troian et al. (3). The normalized gene expression relative to the *sigA* housekeeping gene was calculated based on *ΔΔ*CT. Statistical analyses were performed using an unpaired, two-tailed Student’s *t*-test.

### Antibiotic MIC determination

To determine the minimum inhibitory concentration (MIC) for ciprofloxacin (CIP) and RFB, a 1 mg/mL stock solution of CIP was freshly prepared in dimethyl sulfoxide (DMSO) on the day of the experiment; a 10 mg/mL stock solution of RFB in DMSO was prepared, aliquoted, and stored at −80°C. Mab ATCC 19977 cells were inoculated and grown in 7H9 Middlebrook medium with the supplements and conditions listed in the first methods section. When cells reached an OD_600_ of ∼0.1 (∼1 × 10^7^ CFU/mL), the culture was aliquoted and diluted to 1 × 10^5^ CFU/mL per 100 µL of cells. CIP and RFB were diluted to a final working concentration of 25 and 64 µg/mL, respectively. Negative control wells contained 200 µL of complete 7H9 medium with no antibiotic or cells. Antibiotic wells contained 100 µL of serially diluted antibiotic solution, along with 100 µL of diluted cells. Growth control wells contained 100 µL of complete 7H9 Middlebrook medium without antibiotic and 100 µL of diluted cells. All wells had a total volume of 200 µL prior to sealing and incubating the plates at 37°C without agitation. After the incubation period, 30 µL of 0.015% resazurin was added to the wells and allowed to incubate overnight. The MIC was determined as the antibiotic concentration of the first blue-purple wells in the dilution series as compared to the growth (pink) and negative (blue) controls.

Aziz et al. (9) used 0.39 µM for their RFB subinhibitory MIC; their RFB MIC was 3 µM. Since we obtained an MIC of 2 µg/mL (2.4 µM) for RFB (Supplementary Figure S1), we used a subinhibitory MIC of 0.3 µM [proportionally reduced given our lower MIC compared to that reported by Aziz et al. (9)].

### Persister cell assay

Mab ATCC 19977 containing either empty vector pMC1s (−VapC5) or pMC1s-*vapC5* (+VapC5) was freshly transformed and grown in 7H9 Middlebrook medium with the supplements and conditions listed in the first methods section until the OD_600_ reached ∼0.1. Cultures could be diluted to OD_600_ ∼0.1 from cultures with an OD_600_ ≤ 0.25. Then both −VapC5 and +VapC5 cultures were induced with ATc to a final concentration of 200 ng/mL. After 5–6 h of induction, 1 mL of -VapC5 and 1 mL of +VapC5 cells were aliquoted, serially diluted in 1X phosphate-buffered saline with Tween (PBST), and plated on complete 7H11 + kanamycin plates to determine CFU/mL. Then, 20 µg/mL of CIP (10X MIC) or 100 µg/mL of RFB (50X MIC) was added to each + VapC5 culture that exhibited toxicity and empty vector −VapC5 control cultures. After 24 and 48 h of antibiotic treatment, 1 mL of cells was aliquoted and centrifuged at 15,000×*g* for 2 min. The cells were then washed twice with 1X PBST, serially diluted, and plated on complete 7H11 + kanamycin to determine CFU/mL. The plates were incubated at 37°C for 4–5 days. Cell survival rate was determined by comparing colonies recovered after 24 and 48 h antibiotic treatment to the initial postinduction, pretreatment colony numbers (0 h). Statistical analyses were performed using an unpaired, two-tailed Student’s *t*-test.

As stated in Troian et al. (3), the following guidelines were used to ensure accurate and consistent results: (1) Mab + VapC5 cells must be freshly transformed ∼1 week prior to each experiment; (2) +VapC5 cells must display toxicity when compared to -VapC5 cells after 5–6 h of ATc induction; (3) samples exhibiting antibiotic resistance should be eliminated; (4) consider removing samples exhibiting clear outlier behavior with time relative to other replicates; (5) cells diluted to an OD_600_ ∼0.1 prior to induction must not originate from cultures with an OD_600_ greater than 0.25; (6) colony counts between 20 and 300 within a dilution series are considered significant; and (7) colony counts within a dilution series should be representative of the dilution pattern. Outliers that occasionally occur due to cell clumping can be eliminated.

## Results

### VapC5 selectively activates a distinct subset of the WhiB7 regulon

Our 2023 VapC5 study reported strong increases in transcripts for five of the best characterized members of the WhiB7 regulon, i.e., the *whiB7* transcription factor, the ribosomal methylase *erm(41)*, the Gcn5-related N-acetyltransferase *eis2*, the aminoglycoside 2´-N-acetyltransferase *aac(2’)*, and the *tap* efflux pump (3). However, Hurst-Hess et al. defined a broader Mab WhiB7 regulon in 2017 by identifying 127 transcripts that increased >4-fold upon comparing the *ΔwhiB7* mutant strain to a *ΔwhiB7* mutant strain constitutively expressing *whiB7* (2). In 2023, Hurst-Hess et al. further distinguished direct from indirect WhiB7 targets using chromatin immunoprecipitation followed by sequencing (ChIP-seq), identifying 73 genes whose promoters are directly bound by WhiB7 (1).

We used datasets from these two studies to assess the effect of VapC5 expression on these more comprehensively defined versions of the WhiB7 regulon (Figure 1, Supplementary Table S1). For the 2017 dataset, we converted the >4-fold induction threshold to log_2_ fold change, corresponding to >2 log_2_ fold change, and generated heat maps comparing the 127 WhiB7-responsive transcripts to those after VapC5 induction (Figure 1A). VapC5 increased the steady-state levels of only 90 of 127 transcripts in this dataset (adjusted *p*-value ≤ 0.05 and log_2_ fold change ≥1; Figure 1A, left vs. middle). Moreover, VapC5 expression in the isogenic *ΔwhiB7* deletion mutant markedly dampened the overall induction profile of these transcripts (Figure 1A, right), indicating that much of the VapC5 response requires WhiB7.

**Figure 1:**
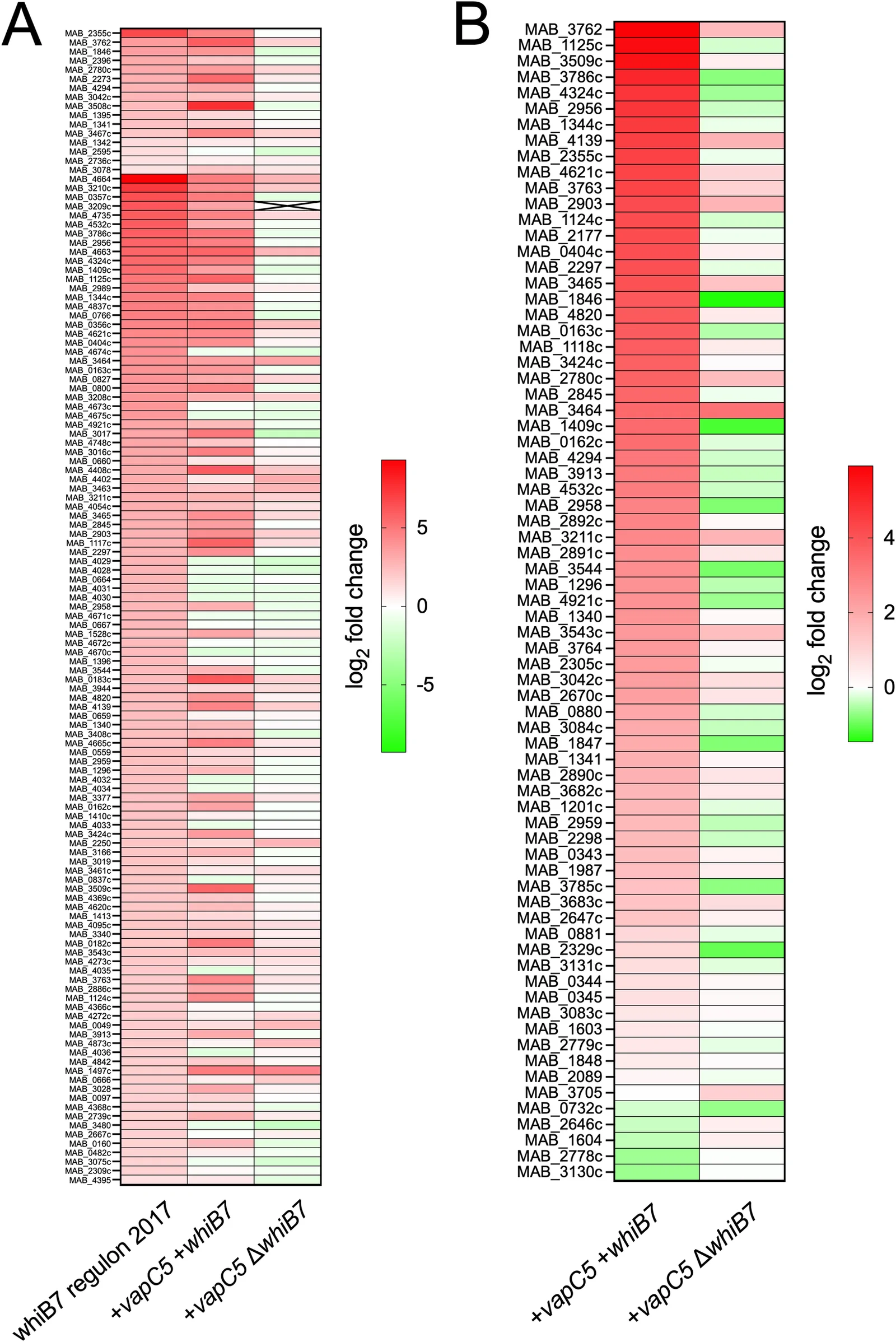
VapC5 enhances expression of a subset of the WhiB7 regulon. **(A)** (left, “WhiB7 regulon 2017”) The 127 transcripts assigned to the WhiB7 regulon were defined upon comparing RNA-Seq datasets from a *ΔwhiB7* strain to a *ΔwhiB7* strain complemented with *whiB7* (2) (middle, “+*vapC5* +*whiB7*”), a wild-type Mab strain expressing VapC5, and (right, “+*vapC5 ΔwhiB7*”) the *ΔwhiB7* strain expressing VapC5. **(B)** A 73-transcript WhiB7 regulon (excluding *whiB7*) defined by ChIP-seq (1) shows the effects of VapC5 expression when *whiB7* is present (left, “+*vapC5* +*whiB7*”) or deleted (right, “+*vapC5 ΔwhiB7*”). All detected transcripts from RNA-Seq are shown in both panels; MAB_3209c, not detected (“X”). Source data with adjusted *p*-values in Supplementary Table S1.

We next assessed how VapC5 influences the expression of the 73 direct WhiB7 target genes identified by ChIP-seq. VapC5 upregulated nearly all genes in this subset of wild-type Mab (Figure 1B, left), whereas VapC5 expression in the *ΔwhiB7* mutant enhanced transcript levels of only 10 of the 73 WhiB7 targets (adjusted *p*-value ≤ 0.05 and log_2_ fold change ≥1; Figure 1B, right). Together, these analyses indicate that VapC5 does not uniformly activate the entire WhiB7 regulon but instead selectively induces a distinct, largely WhiB7-dependent subset of WhiB7-regulated genes.

Finally, in our 2023 study, we showed that transcripts encoding many other WhiB family members of unknown function, and 12 of the 13 antibiotic response genes identified by Miranda-CasoLuengo et al. (10), were upregulated upon VapC5 expression (Figure 2, left). However, these responses were largely absent when VapC5 was expressed in the *ΔwhiB7* mutant (Figure 2, right). Therefore, WhiB7 is also required for VapC5-mediated induction of other WhiB-family and resistance-associated genes.

**Figure 2:**
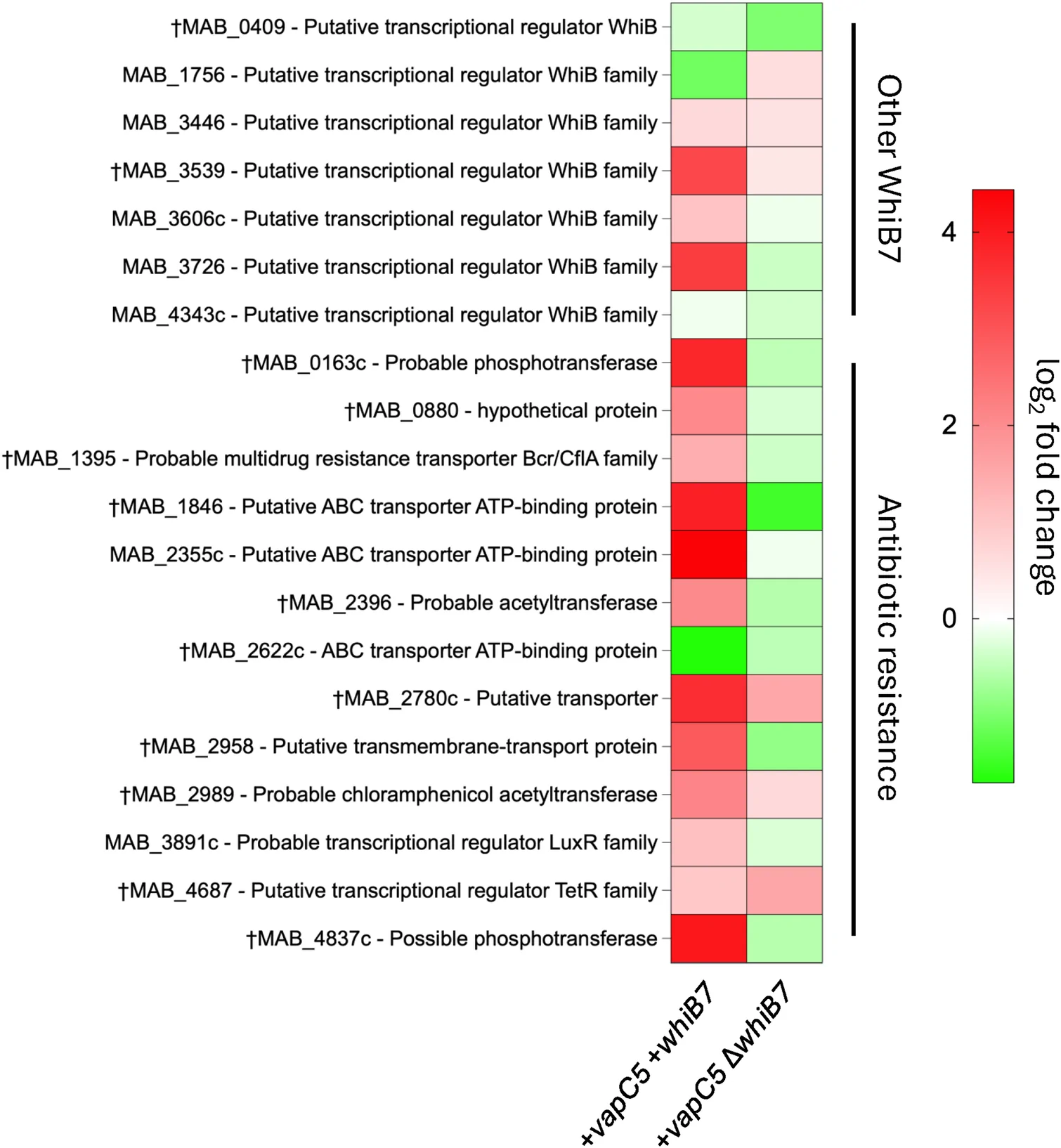
Loss of WhiB7 abrogates the effect of VapC5 on expression of antibiotic resistance determinants and other *whiB7*-family genes. Differential gene expression upon VapC5 expression in the wild-type *whiB7* or *ΔwhiB*7 background. “Other WhiB” indicates transcripts annotated as WhiB-related family members, and “Antibiotic Response” corresponds to 13 transcripts associated with antibiotic resistance identified in Miranda-CasoLuengo et al. (10). Statistically significant transcripts (adjusted *p*-value ≤ 0.05) are indicated by asterisks (*) for the wild-type *whiB7* strain; daggers (†) for the *ΔwhiB7* strain.

### VapC5 activates DNA repair and lipid biosynthetic pathways in *Δ*whiB7 cells

A hallmark of VapC5 toxin expression in wild-type Mab cells is growth arrest (3); this arrest phenotype was maintained when VapC5 was expressed in the *ΔwhiB7* deletion mutant (Figure 3A). However, these growth-arrested *ΔwhiB7* cells no longer resisted clearing by cefoxitin (FOX, Figure 3B) or amikacin (AMK, Figure 3C) as observed previously in the wild-type strain.

**Figure 3:**
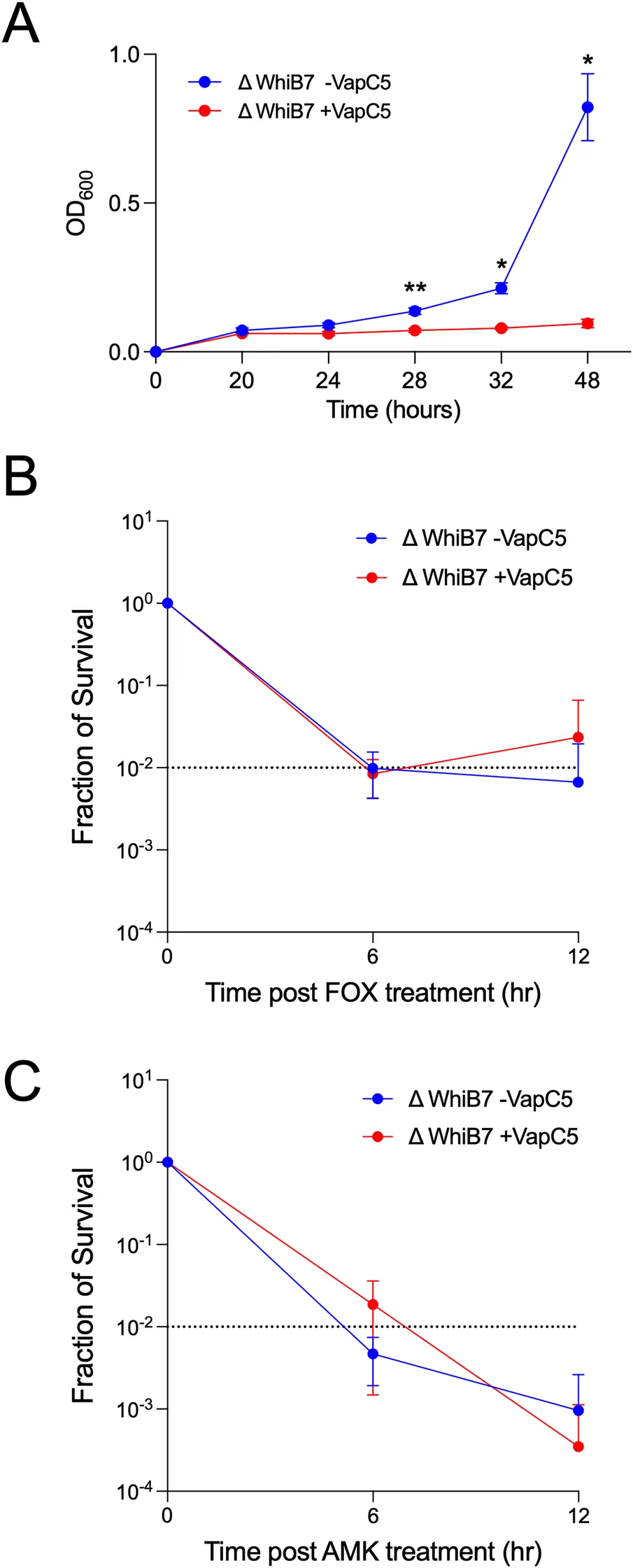
VapC5 arrests growth in *ΔwhiB7* cells but does not lead to persistence after FOX or AMK treatment. (**A**) Mab ATCC 19977:: *ΔwhiB7* cultures harboring either an empty vector (-VapC5, blue) or *vapC5-pMC1s* (+VapC5, red) were grown in 7H9 media and induced with ATc from inoculation. Error bars represent standard deviation from the average of three biological replicates. Asterisks represent statistical significance between the −VapC5 and +VapC5 samples using the unpaired, two-tailed Student's *t*-test (*p ≤ 0.05, **p ≤ 0.01). (**B,C**) Kill curves of *ΔwhiB7* Mab cells with (red) or without (blue) VapC5 expression. 10X MIC was used for FOX **(B)** and AMK **(C)**. Error bars indicate standard deviation; no statistical significance in **B**, **C** using the unpaired, two-tailed Student's *t*-test. Five replicates were used for both antibiotics.

To identify pathways that might contribute to VapC5-mediated toxicity in the absence of WhiB7, we used the DAVID functional annotation tool (11, 12) to determine whether differentially expressed transcripts, enriched or depleted, could be grouped into specific functional categories. Among upregulated transcripts, DAVID identified 18 enriched functional annotation categories. Because many of these categories overlapped or contained identical transcripts such as “DNA damage” and “DNA repair,” we distilled them into two major functional themes: DNA repair and lipid biosynthesis (Figure 4A). No functional categories were enriched among downregulated transcripts. Notably, the *ΔwhiB7* transcriptional profile lacked the striking upregulation of ribosomal protein transcripts observed when VapC5 is expressed in wild-type cells (3). Heat maps of transcripts assigned to the DAVID DNA repair and lipid biosynthesis categories illustrate that these pathways are activated only upon VapC5 expression in the *ΔwhiB7* mutant (Figures 4B,C).

**Figure 4:**
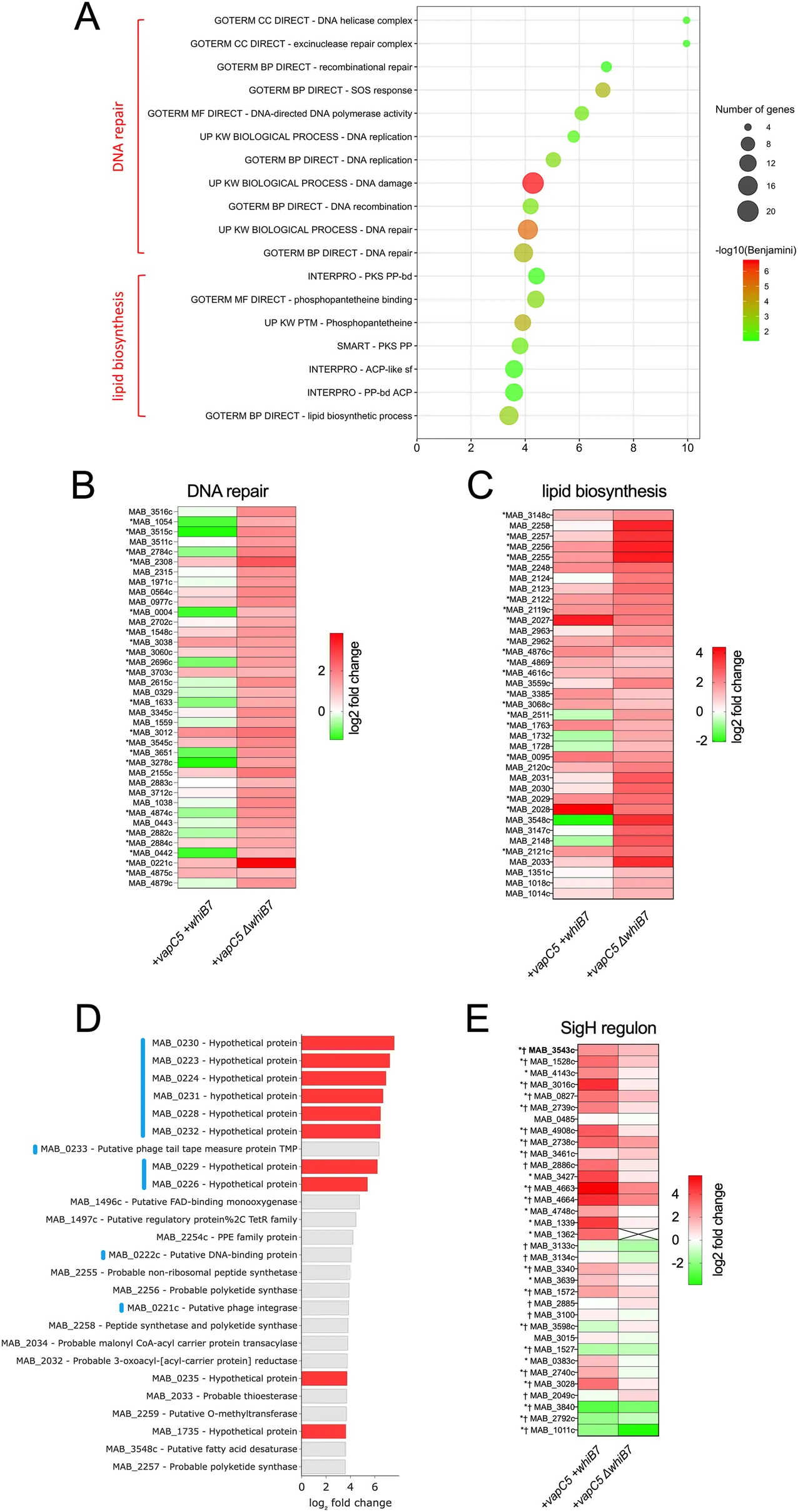
*Δwhib7* cells expressing VapC5 exhibit a maladaptive stress response. **(A)** DAVID analysis (11, 12) of significantly upregulated transcripts (log_2_fold change ≥1, *p* ≤ 0.05) upon VapC5 expression in the wild-type *whiB7* vs. *ΔwhiB*7 background. **(B)** Heat map of the 38 statistically significant transcripts assigned to the DNA repair pathway by DAVID enriched in the +vapC5, +*ΔwhiB*7 strain (right column) compared to the expression level of these transcripts in the +vapC5, + *whiB*7 strain (left column). Statistically significant transcripts in the wild-type *whiB7* strain (adjusted *p*-value ≤ 0.05) are marked with asterisks. **(C)** Heat map of the 37 lipid biosynthesis transcripts identified by DAVID analysis enriched in the +vapC5, + *ΔwhiB*7 strain *(right column) c*ompared to the expression level of these transcripts in the +vapC5, + *whiB*7 strain (left column). Statistically significant transcripts in the wild-type *whiB7* strain (adjusted *p*-value ≤ 0.05) are marked with asterisks. **(D)** Top 25 RNA-Seq hits ranked by descending log_2_ fold change in the *ΔwhiB7* strain expressing the VapC5 toxin. Among these 25 statistically significant transcripts (*p* ≤ 0.05), eight of the top 10 encode hypothetical proteins. Red bars, hypothetical proteins; gray bars, annotated genes. Transcripts derived from clustered genes spanning MAB_0222c through MAB_0233 marked with a blue vertical line. **(E)** Members of the Mab SigH regulon as defined by Hurst-Hess et al. (1) in the +*vapC5,* +*whiB7* vs. +*vapC5 ΔwhiB7* strains. X, gene not detectable in this dataset.

In parallel with the DAVID analysis, we examined the most highly differentially expressed transcripts in *ΔwhiB7* cells (Figure 4D). Eight of the 10 most strongly induced transcripts, which ranged from 7.6 to 4.7 log_2_ fold change, were annotated as hypothetical proteins. Surprisingly, the majority of these highly induced transcripts clustered within a single genomic region spanning MAB_0222c (a divergently transcribed DNA-binding protein) and MAB_0223 through MAB_0233, which also encodes a putative phage integrase and tail tape-measure protein among the hypothetical proteins (Figure 4D). By comparison, only one of these eight hypothetical protein transcripts, MAB_0224, was significantly induced upon toxin expression in wild-type cells, and its induction was markedly lower than in the *ΔwhiB7* mutant (2.1 vs. 6.9 log_2_ fold change in wild-type vs. *ΔwhiB7* cells, respectively). Curiously, the putative phage tail tape measure protein *MAB_0233* has been recently shown to enhance killing by antibiotics that target protein synthesis but not the cell wall (13). Among 405 sequenced clinical isolates tested for in vitro antimicrobial lethality, several deletions of *MAB_0233* were associated with drug tolerance to amikacin, linezolid and tigecycline while MICs were stable (13). These findings indicate that this expression signature is specific to VapC5 in the absence of WhiB7.

Finally, Hurst-Hess et al. reported that the transcriptional regulator SigH was activated by WhiB7 and that SigH induced 33 genes that are not in the Mab WhiB7 regulon (1). Because the SigH regulon responds to heat, oxidative, and nitric oxide stress (1, 14), which are common triggers of TA toxin activity, we assessed how VapC5 expression affected this SigH-associated gene set in the wild-type and *ΔwhiB7* backgrounds (Figure 4E). Loss of WhiB7 significantly dampened the SigH response to VapC5 expression, indicating that WhiB7 is required for full activation of this stress-response signature.

Integrating the findings from Figure 4 suggests that loss of WhiB7 unmasks an alternative VapC5-induced stress state comprising seemingly disparate stress responses that include activation of the SOS/DNA repair response, lipid biosynthesis, and a clustered prophage- or mobile element-associated locus among the most highly expressed transcripts. Mab strains commonly carry prophage-like sequences and mobile genetic elements containing accessory genetic cargo. Thus, the MAB_0222c through MAB_0233 locus may reside within a mobile element, a common feature of the Mab accessory genome. The concomitant activation of the SOS/DNA damage and repair pathways further supports the possibility that the MAB_0222c through MAB_0233 locus contains phage-associated genes, because DNA damage is a classic trigger of prophage induction and lytic activation. Activation of this phage-associated locus in VapC5-expressing *ΔwhiB7* cells could help explain why these growth-arrested cells fail to survive FOX and AMK treatment in persister assays.

### Ciprofloxacin or rifabutin kill Mab despite VapC5 expression in wild-type cells

Knowing the mechanistic details underlying the phenotypic switch caused by the VapC5 toxin in wild-type cells, we evaluated all possible antimicrobial candidates whose mechanism of action did not involve the cell wall or translation machinery. We narrowed down our options by considering only alternative antimicrobials with demonstrated efficacy against Mab (14–16). Unfortunately, the majority of treatment options for Mab target the ribosome or cell wall and were not considered. Of the remaining, several were not suitable due to high cost, low availability, or variable MIC behavior. For example, the recently developed DNA gyrase inhibitor evybactin (17) is prohibitively expensive with long lead times. Another option, clofazimine (a DNA gyrase inhibitor), exhibited variable MICs depending on the day the plate was read and on the nutrient content of the medium, consistent with an earlier published report (18). Finally, the ATP synthase inhibitor bedaquiline also exhibited significant MIC variability (16, 18).

Two antibiotics fit our criteria: the fluoroquinolone CIP, which targets DNA gyrase, and the rifamycin RFB, which targets the RNA polymerase β subunit. However, RFB warrants special consideration because rifamycins are generally less active against Mab due to susceptibility to inactivation by oxidation and by the ADP-ribosyltransferase (Arr) [reviewed in (19)]. Nevertheless, this drug class remains promising because optimized RFB analogs that evade Arr inactivation were recently developed and show superior efficacy in a mouse model (20).

We first determined the MIC in Mab as 2 μg/mL for each antibiotic (Supplementary Figure S1). Then, Mab cells with or without VapC5 expression were exposed to 10X MIC for CIP and 50X MIC for RFB for up to 48 h, serially diluted, and plated to determine CFU/mL. We used several times the MIC as recommended (21) to ensure measurement of true persistence and not transient modes of resistance (Figures 5B,D). In tandem, we measured the fraction of survival by plotting time-kill curves to enable comparison of the effect of VapC5 to the control (Figures 5A,C). The kill curves essentially overlapped for both CIP and RFB, indicating that toxin expression did not reduce the efficacy of either antibiotic (Figures 5A,C) nor did they result in any increase in viable cell recovery, i.e., persisters. This is in stark contrast to the increased persister formation and reduction of killing after exposure to cefoxitin, amikacin, and tedizolid in VapC5-expressing cells (3). Consequently, CIP and future optimized RFB analogs appear to be excellent therapeutic options in lieu of standard frontline antibiotics whose potency is predicted to be abrogated in patients infected with a Mab strain that acquired a genomic copy of the VapBC5 TA system.

**Figure 5:**
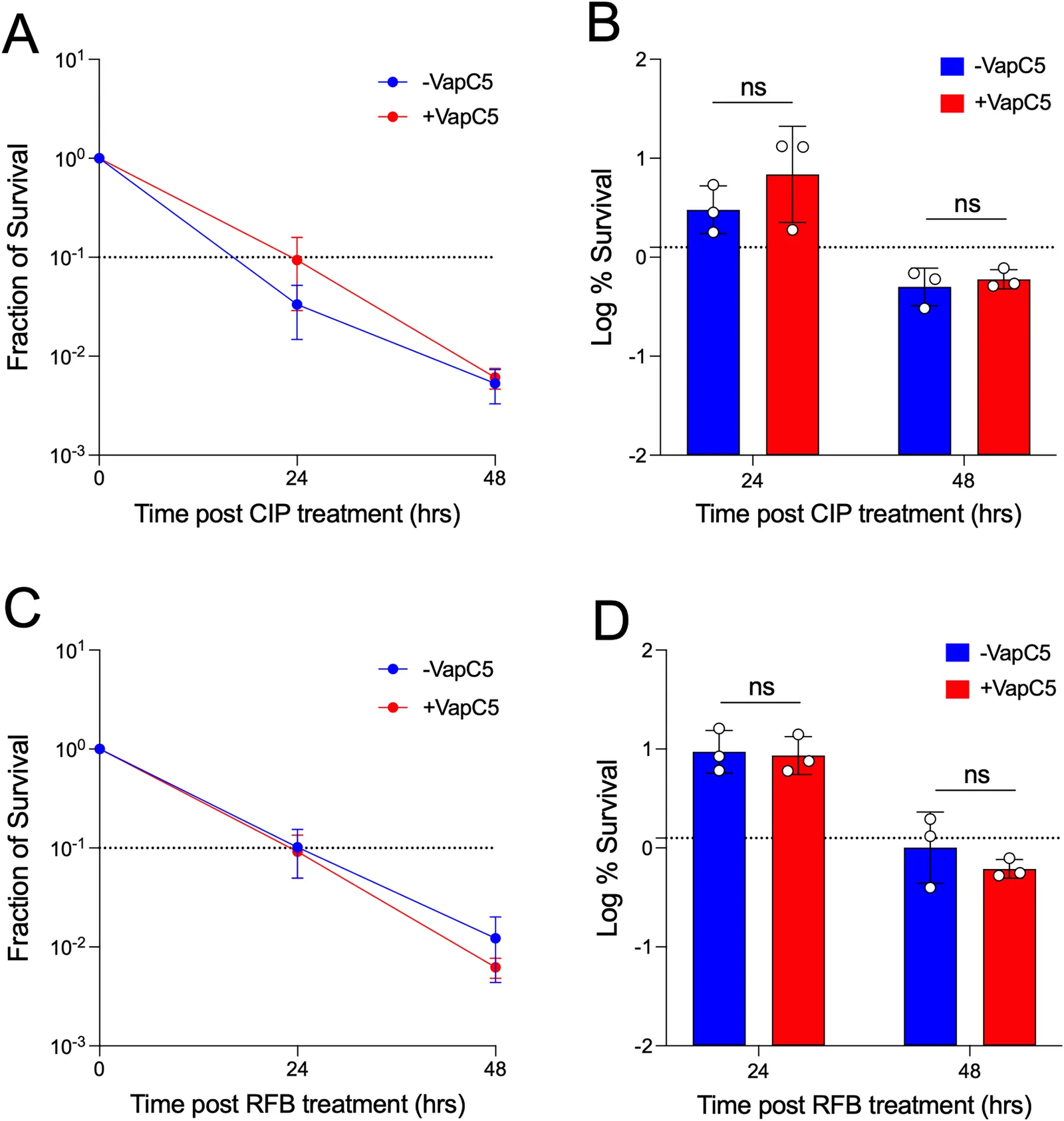
Ciprofloxacin or rifabutin efficiently kill Mab cells expressing the VapC5 toxin. + VapC5 (red), expression of VapC5 in Mab ATCC 19977 induced with ATc; −VapC5, +ATc (blue) for cells carrying an empty vector. Antibiotic concentrations were 10X MIC for CIP **(A,B)** or 50X MIC for RFB **(C,D)**. Error bars indicate standard deviation; ‘ns’, no statistical significance using the unpaired, two-tailed Student's *t*-test. In **B**, 24 h *p* = 0.3377 and 48 h *p* = 0.5751; in **D**, 24 h *p* = 0.8350 and 48 h *p* = 0.4126.

### Rifabutin synergy with clarithromycin is maintained when the VapC5 toxin is expressed in wild-type cells

Although clarithromycin (CLR) is frequently selected for initial treatment of Mab infections, its efficacy is often compromised because many strains harbor the unmutated allele of the inducible *erm41* ribosome methylase gene associated with resistance to CLR (22, 23). The use of optimized RFB as a frontline drug has the advantage of synergy with CLR and other ribosome-inactivating antibiotics (9, 24, 25). Mechanistically, the Dick laboratory reasoned that since RFB inhibits RNA polymerase activity, it should then block activation of the *whiB7* transcription factor gene and thus also prevent upregulation of the *whiB7* regulon members (1, 2), including *erm41* (9). Indeed, RFB treatment of any of the six CLR-resistant Mab strains (including the ATCC 19977 reference strain we use for our VapC5 studies) resulted in CLR sensitivity because transcription of both the master regulator *whiB7* and its target *erm41* was inhibited, as assessed by qRT-PCR (9). As a result, the authors propose that the inclusion of RFB as a frontline partner in combination therapy against Mab infections should be investigated in therapeutic trials. These results linking *whiB7* transcript levels to its downstream target genes are consistent with another study using a Mab *whiB7* deletion strain (25). Using this strain, the authors quantified two additional *whiB7* regulon members known to contribute to intrinsic resistance, i.e., *eis2*, encoding an efflux pump and *aac(2’)*, encoding an aminoglycoside 2'-N-acetyltransferase. Both *eis2* and *aac(2’)* transcripts were profoundly downregulated upon CLR treatment in this *whiB7* deletion strain (25).

Since VapC5 expression also results in significant upregulation of the Mab *whiB7* gene in the absence of antibiotic treatment (3), we tested whether RFB treatment could also override *erm41* expression (Figure 6). We quantified *erm41* and *whiB7* transcript levels in cells expressing VapC5 with or without RFB treatment (Figure 6). There was no statistically significant difference in *erm41* levels in − VapC5 vs. + VapC5 cells treated with sub-MIC concentrations of RFB (9) (Figures 6B,C). Therefore, VapC5 activation of *whiB7* transcription does not perturb the RFB-CLR synergy reported by other groups because *erm41* and *whiB7* transcript levels remain low upon RFB treatment of VapC5-expressing cells.

**Figure 6:**
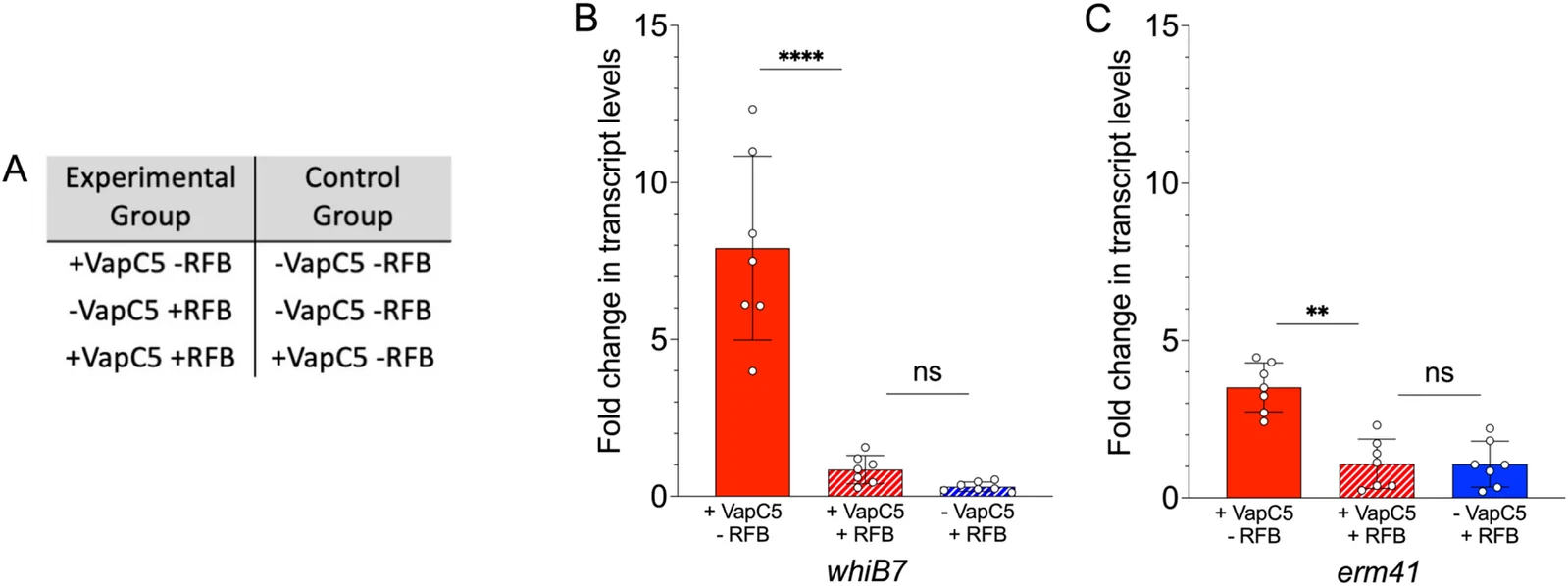
Rifabutin treatment of VapC5-expressing cells decreases *whiB7* and *erm41* transcript levels in a manner comparable to cells without toxin expression. **(A)** A table shows the samples used to calculate the fold changes of *whiB7* and *erm41* expression. **(B)** Subinhibitory concentrations of RFB (0.25 *μ*g/mL) significantly decrease *whiB7* expression when VapC5 is expressed [compare +VapC5/−RFB (solid red) to +VapC5/+ RFB (red hatched)]. This significant reduction in *whiB7* transcript levels is similar to the effect of RFB on cells that do not express VapC5 [compare + VapC5/+ RFB (red hatched) to −VapC5/+RFB (blue hatched)] as reported by Aziz et al. (9). **(C)** Likewise, subinhibitory concentrations of RFB in the presence of VapC5 [compare + VapC5/- RFB, (solid red) to + VapC5/+ RFB (hatched red)] reduce *erm41* levels. This reduction in *erm41* is analogous to that reported by Aziz et al. (9) with RFB treatment only [compare + VapC5/+ RFB (red hatched) to −VapC5/+RFB (blue hatched)]. *SigA* was used as an internal control. Error bars indicate standard deviation. Asterisks represent statistical significance (*p* < 0.01) using the ratio-paired, two-tailed Student's *t*-test (****p < 0.0001, **p = 0.0014); “ns”, not significant; *p* = 0.0164 for *whiB7*, *p* = 0.9742 for *erm41*.

## Discussion

These results refine our understanding of VapC5 by uncovering a strong dependence on WhiB7 for the full complement of phenotypes that potentiate evasion of antibiotic killing. In wild-type Mab, VapC5 cleaves tRNA^SerGCA^, arrests growth, and activates WhiB7. WhiB7—highly conserved in the Mab core genome (Supplementary Table S2)—then helps channel the toxin response into a more protective physiological state characterized by induction of resistance-associated genes, ribosome-associated remodeling, and broader stress-response activation. This state allows VapC5-expressing cells to better survive FOX and AMK.

In the *ΔwhiB7* background, VapC5 still imposes its primary stress, cleavage of tRNA^SerGCA^, and growth arrest is maintained. However, these cells can no longer mount the WhiB7-dependent protective program. Instead, *ΔwhiB7* cells expressing VapC5 exhibit a maladaptive stress response characterized by DNA repair/SOS-like activation, lipid biosynthetic remodeling, activation of a prophage/mobile element-associated locus, and a dampened SigH response. Together, these findings suggest that loss of WhiB7 redirects VapC5-induced translational stress toward a damaged or maladapted physiological state rather than the drug-protective persistent state observed in wild-type Mab.

Mab treatment regimens frequently fail because its accessory genome has incorporated a daunting array of poorly defined resistance mechanisms; our 2023 study added the VapBC5 TA system to this armamentarium (3). Since frontline antibiotics AMK and FOX are compromised by VapC5 *in vitro*, we previously proposed PCR screening for the presence of VapBC5 to guide the course of treatment [much easier than the DNA sequencing used to detect the *erm41* gene and *rrl* mutations associated with CLR resistance (26)]. Here, we identify alternative treatment options for clinical Mab strains to be used as a foundation for further study, as summarized in Figure 7. These *in vitro* studies suggest that CIP and optimized RFB analogs represent an excellent starting point for the development and implementation of effective treatment alternatives in Mab clinical strains harboring the VapBC5 TA system.

**Figure 7:**
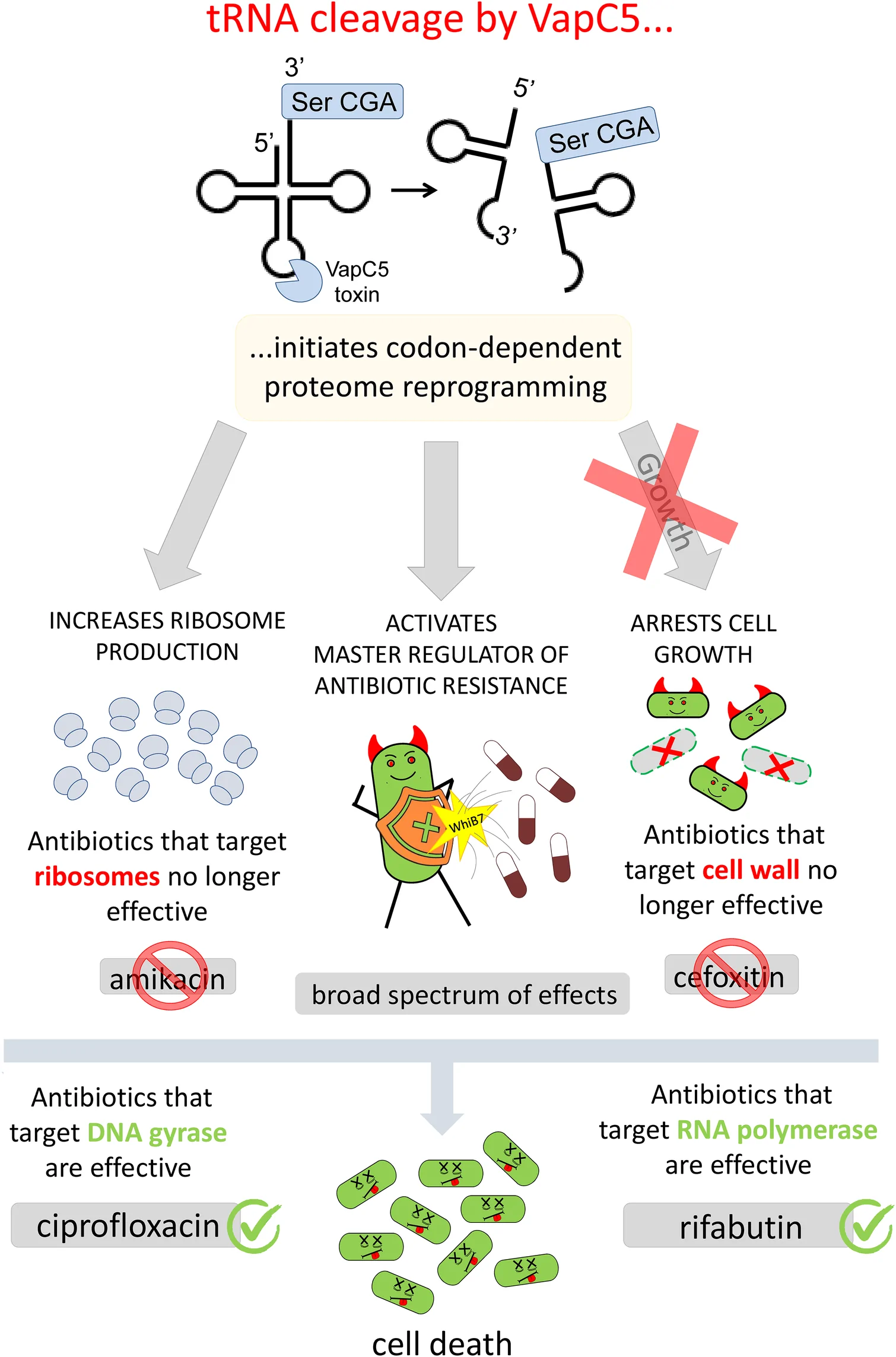
Understanding how VapC5 is predicted to sabotage frontline regimens can guide selection of more effective treatment options for clinical Mab strains. The VapC5 endonuclease TA toxin primarily cleaves and depletes the serine tRNA that services the most abundant serine codon UCG in Mab (3). This change in tRNA availability leads to ribosome stalling at UCG codons, triggering transcriptional changes that remodel the Mab proteome toward (1) robust, sustained, and nearly exclusive synthesis of ribosomes; (2) upregulation of *whiB7* and its regulon, shown here to be essential for the full potency of VapC5; and (3) a state of cell growth arrest. The overproduction of ribosomes dampens the efficacy of antibiotics that target ribosomes, and cells in a growth-arrested state (“X” cells) are invulnerable to antibiotics requiring actively growing cells (surviving green cells). Antibiotics such as CIP and RFB target pathways that are not affected by VapC5 and lead to cell death *in vitro*. Consequently, upon future *in vivo* validation of our findings and the development of better RFB analogs, PCR screening to identify VapC5-containing strains should enable clinicians to redirect treatment regimens away from those most recommended, i.e., clarithromycin with amikacin plus cefoxitin or imipenem (27), to those that target pathways not affected by this toxin (DNA gyrase or RNA polymerase inhibitors).

## Data Availability

The RNA sequencing data generated in this study have been deposited in the NCBI Sequence Read Archive under accession number PRJNA1483671.

## Supplementary material

Supplementary Figure 1 MIC determination for CIP and RFB. For CIP (left), +Mab −CIP is the positive control, with 105 cells/mL of Mab ATCC 19977 without CIP; −Mab, −CIP is the negative control, with complete 7H9 media only without CIP. The MIC for CIP was 1.56 µg/mL and rounded up to 2 µg/mL. For RFB (right), +Mab −RFB is the positive control, with 105 cells/mL of Mab ATCC 19977 without RFB; −Mab −RFB is the negative control, with complete 7H9 media only without RFB. The MIC for RFB is 2 µg/mL.
